# Simplified Aeroelastic Model for Fluid Structure Interaction between Microcantilever Sensors and Fluid Surroundings

**DOI:** 10.1371/journal.pone.0123860

**Published:** 2015-04-21

**Authors:** Fei Wang, Liang Zhao, Yanling Zhang, Zhi Qiao

**Affiliations:** School of Mechanical and Electronic Engineering, Harbin Institute of Technology, Harbin, 150001, China; Tsinghua University, CHINA

## Abstract

Fluid-structural coupling occurs when microcantilever sensors vibrate in a fluid. Due to the complexity of the mechanical characteristics of microcantilevers and lack of high-precision microscopic mechanical testing instruments, effective methods for studying the fluid-structural coupling of microcantilevers are lacking, especially for non-rectangular microcantilevers. Here, we report fluid-structure interactions (FSI) of the cable-membrane structure via a macroscopic study. The simplified aeroelastic model was introduced into the microscopic field to establish a fluid-structure coupling vibration model for microcantilever sensors. We used the finite element method to solve the coupled FSI system. Based on the simplified aeroelastic model, simulation analysis of the effects of the air environment on the vibration of the commonly used rectangular microcantilever was also performed. The obtained results are consistent with the literature. The proposed model can also be applied to the auxiliary design of rectangular and non-rectangular sensors used in fluid environments.

## Introduction

Most mechanical measurements using atomic force microscopy (AFM) have been conducted in fluid environments, such as the the non-destructive calibration method for spring constants of microcantilevers based on its frequency response immersed in air[1–5]. It was observed that the microcantilever sensor has a significant frequency shift in the air which can not be interpreted by only damping, resulting in the difficulty on establishing an accurate mode reflecting the relationships between the resonant frequency of a micro-cantilever sensor in the air and its physical properties such as stiffness or Young’s modulus et., al [4–7]. Therefore, since cantilever-type micro-force sensors are mainly used in the air, these disadvantages have greatly limited the accurate force calibration for them, which has limited the development of microcantilever sensor based force measurement technologies at the nanonewton level. Generally, the cantilever microstructure has been widely used in the field of micro-electromechanical systems (MEMS), in which the frequency response test of this structure in air is usually applied to obtain the desired parameters [8–13].

Currently, many researchers are paying close attention to this phenomenon. However, the cause of the abnormal reduction of the resonant frequency of cantilever-type micro-force sensors in air remains controversial. Some researchers have attributed this phenomenon to fluid structure interaction (FSI) [4,5], while others have attributed it to surface effects. In a study that considered cantilever-type micro-humidity sensors, Shih *et al* [6,7], observed anomalous resonant frequencies and attributed them to surface effects. These authors believed that the surface-adsorbed water molecules of the micro-beam caused changes in the surface stress and variations in the Young's modulus that resulted in the resonant frequency changes. In a study concerning the stiffness calibration of cantilever-type micro force sensors that were used in AFM, Sader *et al*. noticed the previously mentioned aberrant reductions in resonant frequency and attributed them to FSI [4]. These authors established a one-dimensional FSI model for uniform rectangular cross-section micro-cantilever sensors with high Reynolds numbers and proposed a stiffness calibration method that accounts for the FSI. Nevertheless, Sader’s method is not suitable for non-rectangular micro-cantilever sensors because the theoretical solution on differential equations in the FSI model for non-rectangular micro-cantilever sensors is unfeasible. Although it is unknown whether the FSI are a major cause of the cantilever’s aberrant resonant frequency, the occurrence of FSI between micro-cantilever sensors and the fluid environment as well as their impacts on the dynamic properties of micro-cantilevers have generally been recognized in academia.

A number of scholars have studied the physical phenomenon of FSI at a macroscopic scale [14–18]. However, due to the complexity of the mechanical properties of the micro-cantilever and insufficient high-precision microscopic mechanical measurement instruments, exploring the FSI of micro-cantilevers is more challenging than exploring other macroscopic structures. Existing classical analytical formulas are not applicable for investigating the FSI of micro-cantilevers, especially for non-rectangular micro-cantilever beams, such as triangular micro-cantilever beams. In addition, cable-membrane structures (macrostructure) have mechanical properties that are similar to the mechanical properties of micro-cantilevers. For example, both of these structures are ‘light’ and ‘supple’, which results in distinct FSI of the cable-membrane structures in air. Therefore, according to previous research involving the FSI of macroscopic cable-membrane structures, we introduce a simplified aeroelastic model. This model is generally used to study the FSI of macroscopic cable-membrane structures and to investigate the FSI of micro-cantilever sensors microscopically.

Here, we simplify the vibrations of micro-cantilevers in a fluid environment into a damped forced vibration problem of spring-mass systems and derive an equation for calculating the frequency drift of the micro-cantilevers in a fluid environment by first considering the damping force rather than the coupled fluid-solid vibrations. Next, we model the FSI of the micro-cantilevers with an aeroelastic modeling method and conduct simulation analyses. The simulation results demonstrate that the fluid environment significantly affects the dynamic responses of the micro-cantilevers. This finding is consistent with previous results and is explained by the aeroelastic model proposed in this paper.

## An Equivalent One-Dimensional Vibration Model

**Fig 1:**
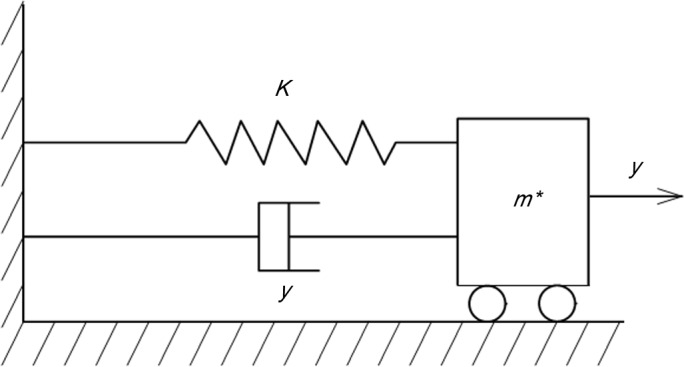
Effective one-dimensional model for cantilever vibration in air.

The differential equation for the vibrations of the micro-cantilevers is simplified into an equation for one-dimensional force vibration. As shown in Fig 1, the vibration differential equation based on the principles of vibration mechanics and assuming that the fluid damping force on the micro-cantilever beam is proportional to the velocity can be expressed as follows:

$$\ddot{y}+2\delta \dot{y}+\omega_{0}^{2}y=\frac{F_{0}}{m^{*}}e^{i\omega t}$$ 1

where *δ* is given as *δ* = *b*/2*m* ^*^, *b* is the damping coefficient, m* is the equivalent mass, and *ω*_0_ is the angular vibration frequency, which is given as $\omega_{0}^{2}=K/m^{*}$.

The displacement of the free edge can be obtained from *y* = *A* (*ω*) *e* ^*i* (*ωt*-*δ*)^, and the vibration amplitude is given by,

$$A(\omega )=\frac{F_{0}/m^{*}}{\sqrt{{\left(\omega_{0}^{2}-\omega^{2}\right)}^{2}+{\left(2\delta \omega \right)}^{2}}}$$ 2

In addition, the phase angle is given as follows:

$$\tan(\theta )=2\delta \omega /(\omega_{0}^{2}-\omega^{2})$$ 3

Furthermore,

$$\omega_{d}{}^{2}=\omega_{0}^{2}-\delta^{2}$$ 4

resonance occurs with its maximum amplitude given by Eq (5).

$$A_{\max}=\frac{F_{0}Q}{K\sqrt{1-(1/4Q^{2})}}$$ 5

The quality factor is given as follows:

$$Q=\frac{\omega_{0}}{2\delta }$$ 6

Substituting Eq (6) into (4) provides the ratio of the natural vibration frequency in the fluid environment to the natural vibration frequency in the vacuum environment as shown below.

$$\frac{\omega_{d}}{\omega_{0}}=\sqrt{1-\frac{1}{4Q^{2}}}$$ 7

**Fig 2:**
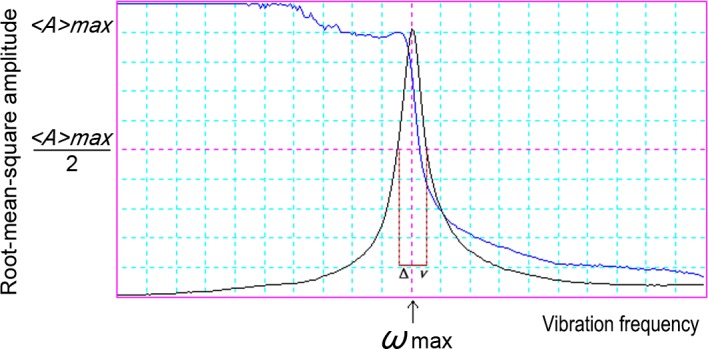
Root-mean-square amplitude curves as a function of frequency for a one-dimensional oscillator with damping.

According to the study of G.Y. Chen, *δ* and *Q* can be derived as follows [19]. First, the vibration response curve of the microcantilever in air is experimentally measured. As displayed in Fig 2, the X-axis denotes the vibration frequency and the Y-axis denotes the root-mean-square amplitude. Next, *δ* and *Q* are calculated based on Eqs (8)(9).

$$Q=\sqrt{3}\frac{v_{\max}}{\Delta v}$$ 8

$$\Delta v=\frac{\sqrt{3}\delta }{2\pi }$$ 9

Here, *v*_max_ is the resonant frequency of the vibration of the micro-cantilever force by the fluid. In addition, Δ*v* is the frequency deviation that corresponds to a 50% reduction in the root-mean-square amplitude of the microcantilever.

Substituting the derived *δ* value into Eq (6) provides the natural vibration frequency (*ω*_0_) of the micro-cantilever in a vacuum environment.

**Fig 3:**
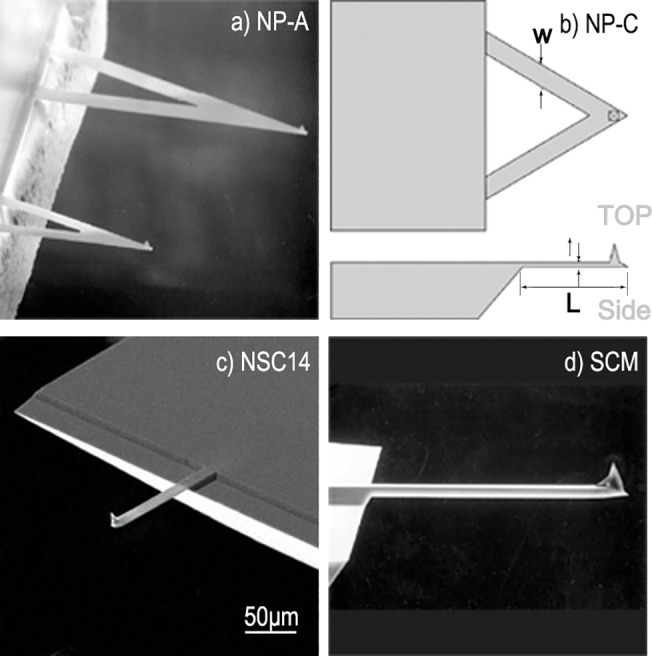
Pictures and diagrams of the AFM cantilevers: a) NP-A b) NP-C c) NSC14 d) SCM.

**Fig 4:**
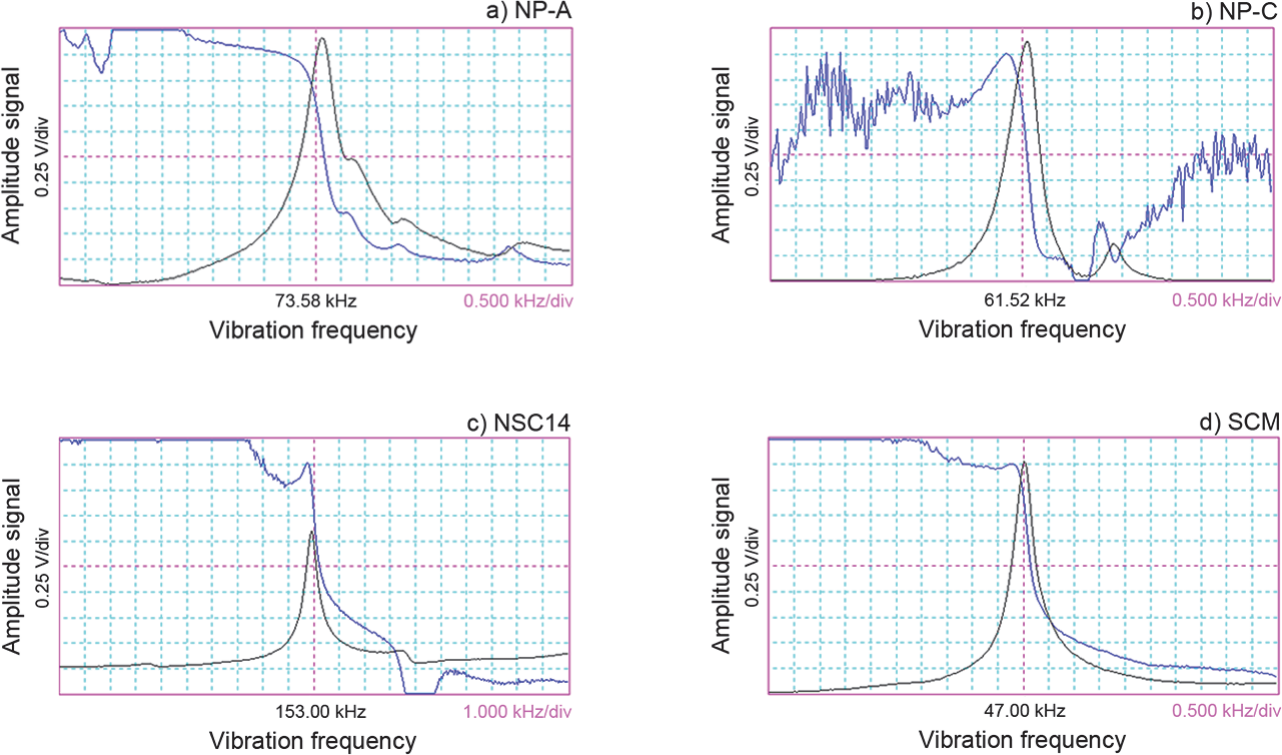
Frequency responses of four common AFM cantilevers: a) NP-A b) NP-C c) NSC14 d) SCM.

Based on Eq (7), we used four commonly used micro-cantilevers as examples for analyzing the impacts of an air environment on the natural vibration frequencies of micro-cantilevers by considering damping forces without FSI. Fig 3A shows the scanning electron microscope (SEM) image of a NP-A micro-cantilever, which is made of silicon nitride and is 115 μm long, 25 μm wide, and 0.6 μm thick. Fig 4A shows the frequency response curve of this cantilever in the air. The NP-C micro-cantilever has dimensions and a structure that are similar to the NP-A micro-cantilever. This micro-cantilever is made of silicon nitride and is 115 μm long, 17 μm wide, and 0.6 μm thick (as shown in Fig 4B). Fig 3C shows the SEM image of an NSC14 micro-cantilever that is made of silicon and is 125 μm long, 35 μm wide, and 2 μm thick. Fig 4C shows the frequency response curve of this cantilever in the air. Fig 3D demonstrates the SEM image of a SCM micro-cantilever that is made of silicon and is 450 μm long, 50 μm wide, and 2 μm thick. Fig 4D displays the frequency response curve of this cantilever in the air. The NP-A, NP-C, and SCM micro-cantilevers were manufactured by the Veeco Company, and the NSC14 cantilever was manufactured by the MikroMasch Company.

According to Eq (7), the *ω*_*d*_/*ω*_0_ values of the NP-A, NP-C, NSC, and SCM micro-cantilevers were 0.99996, 0.99998, 0.99999, and 0.99997, respectively. These results indicate that the air environment barely affects the micro-cantilever vibrations (<1‰). Consequently, the environmental impacts seems negligible. However, in practice rather than the calculated results confirmed that the air exerted a significant impact on the natural vibration frequencies of the micro-cantilevers. Thus, a relatively large discrepancy occurred between the calculation results that were derived from the simplified analysis method and the actual measured results. Therefore, the calculation method that only considers the air damping force is insufficient for ensuring the accuracy of the results. Due to the small dimensions and mass of the micro-cantilever, we believe that the fluid force acting on the micro-cantilever is related to multiple factors when the micro-cantilever is vibrating in a fluid environment. This force is not only proportional to the velocity but also related to acceleration and displacement during physical movement. Thus, the coupled vibration occurs between the micro-cantilever and the air.

## A Simplified Aeroelastic Model

### The model

A simplified aeroelastic modeling method is used to describe the FSI with added mass and damping, whose values can be determined using approximate analytic theories, aeroelastic modeling experiments, or simulation methods.

First, we simplified the micro-cantilever vibration problem to a spring-mass system vibration problem. In this case, the spring-mass system used an equivalent concentrated mass (M) that acted on the end of the micro-cantilever. According to the principles of vibration mechanics, the differential equation for the free vibration of this system is given as follows:

$$M\ddot{x}+kx=0$$ 10

where *k* is the stiffness of the micro-cantilever.

In addition, the natural frequency of this system is represented as follows:

$$\omega_{0}=\sqrt{\frac{k}{M}}$$ 11

The differential equation of motion for the micro-cantilever under the combined action of the external excitation source and the surrounding fluid is given as follows:

$$M\ddot{x}+kx=F_{s}(t)+F_{f}[t,x(t),\dot{x}(t),\ddot{x}(t)]$$ 12

where *F*_*S*_ (*t*) is the external excitation force and *F*_*f*_ is the excitation force produced by the fluid.

Under normal conditions, the response amplitude of a micro-cantilever is much smaller than its structural dimensions. Consequently, the hydrodynamic effects approximately satisfy the standard assumption. This standard assumption suggests that the fluid force acting on a structure is related to the rigid-body configuration and the relative velocity during the moment of action, but not before the motion. Based on this assumption, motion only affects the fluid pressure at the surface during the moment of action and not the vibration characteristics of the flowing fluid.

Therefore, the aerodynamic forces on the right hand side of Eq (12) can be separated to obtain the following equation:

$$M\ddot{x}+kx=F_{s}(t)+p(t)+f[x(t),\dot{x}(t),\ddot{x}(t)]$$ 13

where *p* (*t*) is the hydrodynamic force produced by the pulsation of the fluid itself.

According to Eq (13), if we neglect the effects of acceleration and the velocity of the solid on the excitation force produced by the fluid, the problem can be simplified into the previously mentioned damped vibration problem of spring-mass systems. However, as discussed above, considerable discrepancies occur between the results derived from the frequency response analysis of micro-cantilevers in a fluid environment using a simplified damped vibration model of spring-mass systems and the results obtained from actual experimental measurements. Thus, the effects of the displacement, velocity, and acceleration of the solid body should be considered. Due to the tremendous difficulties involved in theoretically decoupling $f[x(t),\dot{x}(t),\ddot{x}(t)]$ based on a generalized simplification principle in the analysis of FSI at the macroscopic scale, we consider that the fluid force is linearly correlated with displacement, velocity, and solid acceleration as follows [20]:

$$f\left[x(t),\dot{x}(t),\ddot{x}(t)\right]=m_{a}\ddot{x}(t)+c_{a}\dot{x}(t)+k_{a}x(t)$$ 14

Here, rather than a strict analytical formula, Eq (14) is an approximate formula that is generally accepted when solving FSI problems at a macroscopic scale. Here, we introduce Eq (14) for the microscopic analysis because the FSI of the micro/nano-structures is physically consistent with the coupling of the macrostructures. Both methods involve the coupling process in which the vibration of a solid in the fluid environment causes vibrations in the surrounding fluid, which in turn act on the solid. Due to the small size and weight and low stiffness of the micro/nano-structures, coupled fluid-solid vibration often occurs as the micro/nano-structure is vibrating. Therefore, although the values of *m*_*a*_ and *k*_*a*_ in Eq (14) are very small, their effects during the vibration of the micro/nano-solid should be considered due to the small mass and stiffness of the micro/nano-solid bodies.

Substituting Eq (14) into Eq (13) yields the following expression:

$$\left(M+m_{a})\ddot{x}+c_{a}\dot{x}+(k+k_{a})x=F_{s}(t)+p(t\right)$$ 15

Eq (15) indicates that the effects of the fluids on the solids are equivalent to the forced vibration of a spring-mass system loaded with the added mass (*m*_*a*_), damping force (*c*_*a*_), and stiffness (*k*_*a*_). According to the principles of vibrational mechanics, the resonant frequency (*ω*_*a*_) of the system described by Eq (15) is given as follows:

$$\omega_{a}=\sqrt{\frac{k+k_{a}}{M+m_{a}}\left(1-\frac{c_{a}{}^{2}}{2k(M+m_{a})}\right)}$$ 16

Dividing Eq (16) by Eq (11) yields the following expression:

$$\frac{\omega_{a}}{\omega_{0}}=\sqrt{\frac{M}{M+m_{a}}\frac{M\omega_{0}{}^{2}+k_{a}}{M\omega_{0}{}^{2}}\left(1-\frac{c_{a}{}^{2}}{2M\omega_{0}{}^{2}(M+m_{a})}\right)}$$ 17

From Eq (17), if the added mass (*m*_*a*_), damping force (*c*_*a*_), and stiffness (*k*_*a*_) can be determined, the equation of the relationship between *ω*_*a*_ and *ω*_0_ can be obtained, and *ω*_0_ can be derived from the experimentally measured *ω*_*a*_.

### Fluent-assisted measurement of the added fluid parameters

The added fluid parameters can generally be experimentally measured. For easy simulation analysis, we introduce an efficient simulation method in which the computational fluid dynamics software Fluent is applied to acquire the desired fluid parameters.

First, the fluid force on the micro-cantilever at the boundary [from Eq (13)] is equivalent to the combined effects of the added mass, damping force, and stiffness. Therefore, we consider the fluid as a damped spring-mass system. To simplify the description of this system, the fluid force is written as shown below:

$$f_{fluid}=k_{{}_{fluid}}x+c_{{}_{fluid}}v+m_{{}_{fluid}}a$$ 18

Where *f*_*fluid*_ is the interaction between the fluid and the solid, *k*_*fluid*_ is the stiffness of the equivalent spring of the fluid (the added stiffness), *c*_*fluid*_ is the equivalent damping coefficient of the fluid (the added damping), *m*_*fluid*_ is the equivalent mass of the equivalent dampened spring-mass system (the added mass), *x* is the boundary displacement, *v* is the velocity of the solid, and *a* is the acceleration of the solid.

**Fig 5:**
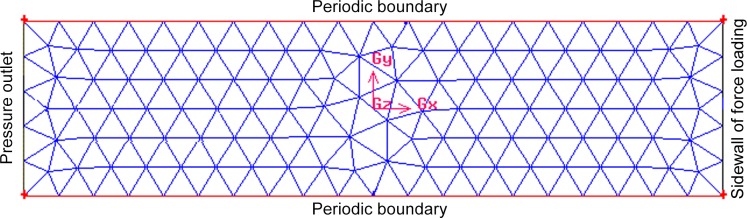
Fluid model.

To solve the added stiffness, damping, and mass, we establish a fluid model using the dynamic mesh model in Fluent (as displayed in Fig 5). The beam moves along the direction of sidewall of force loading to pressure outlet. Next, we apply this model to determine the *k*_*fluid*_, *c*_*fluid*_, and *m*_*fluid*_ parameters according to the procedures described below.

### (1) The added stiffness and damping

**Fig 6:**
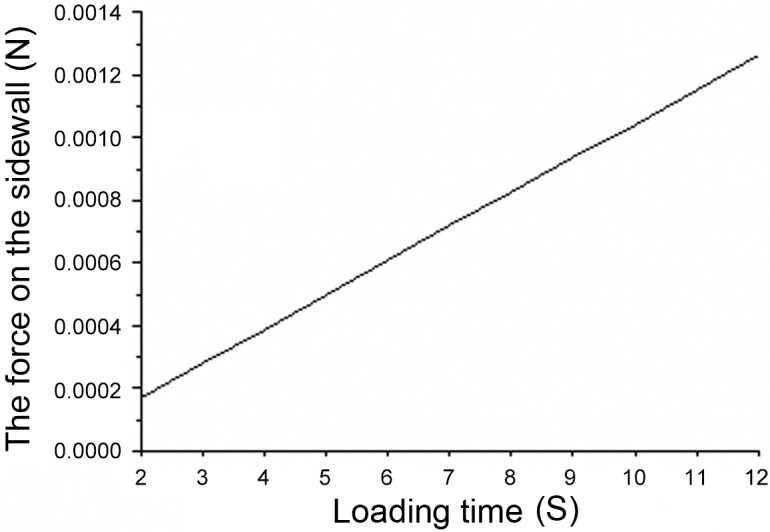
Plot of the force on the edge loaded by a constant speed *v* versus time.

We let the acceleration of the fluid at the boundary equal zero and the velocity of the fluid loaded at the boundary equal v0. Next, we calculate the relationship curve of the fluid force and the force-bearing duration as shown in Fig 6. By multiplying the X-axis values with the velocity at the boundary (v0), we obtain the changing curve of fluid force (ffluid) and the displacement of the solid (x). As indicated by Eq (18), the slope of this curve denotes the added stiffness, and the intercept of this curve divided by the velocity at the boundary (v0) denotes the added damping.

### (2) The added mass

We let the acceleration of the fluid loaded at the boundary equal *a*_0_ and then calculate the relationship curve for the fluid force and the force-bearing duration. Because the initial velocity is zero, the corresponding boundary displacement at a time of *t* [*x* (*t*)] can be expressed as *a*_0_ *t* ^2^/2. Thus, we can obtain the changing curve for the fluid force (*f*_fluid_) relative to the displacement of the solid (*x*). Because the initial velocity is zero (namely, *cv* (0) = 0), the intercept of this curve is *b* = *m*_*fluid*_ *a*_0_, which can be divided by *a*_0_ to derive the added mass [Eq (17)].

## Examples and Analyses

Using the rectangular micro-cantilever as an example, we investigated the effects of air environments on the vibrations of a micro-cantilever as described above. The dimensions of the rectangular micro-cantilever that was used in the simulation test were as follows: length (*L*) = 260 μm, width (*W*) = 51 μm, and thickness (*h*) = 1.69 μm. The properties of the material were as follows: young's modulus (*E*) = 169 GPa, Poisson's ratio (*μ*) = 0.3, and density (*ρ*) = 2330 kg/m^3^. Because the density of air is much lower than the density of the micro-cantilever, the added mass was negligible.

**Fig 7:**
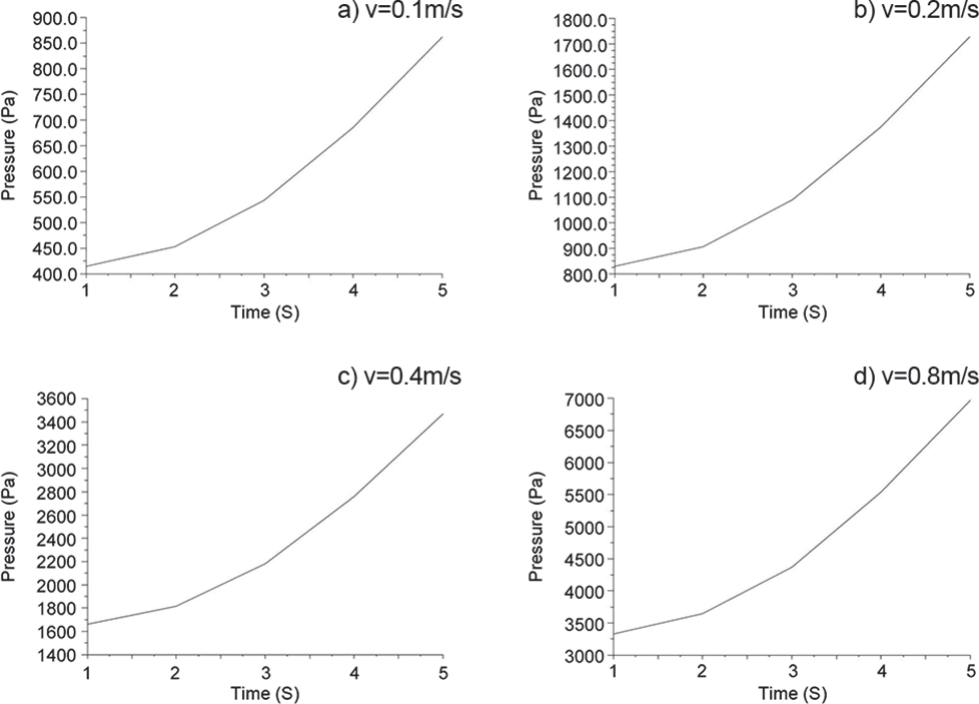
Plot of the force on the edge loaded by different speeds (*v*) versus time:a) v = 0.1m/s b)v = 0.2m/s c)v = 0.4m/s d)v = 0.8m/s.

**Fig 8:**
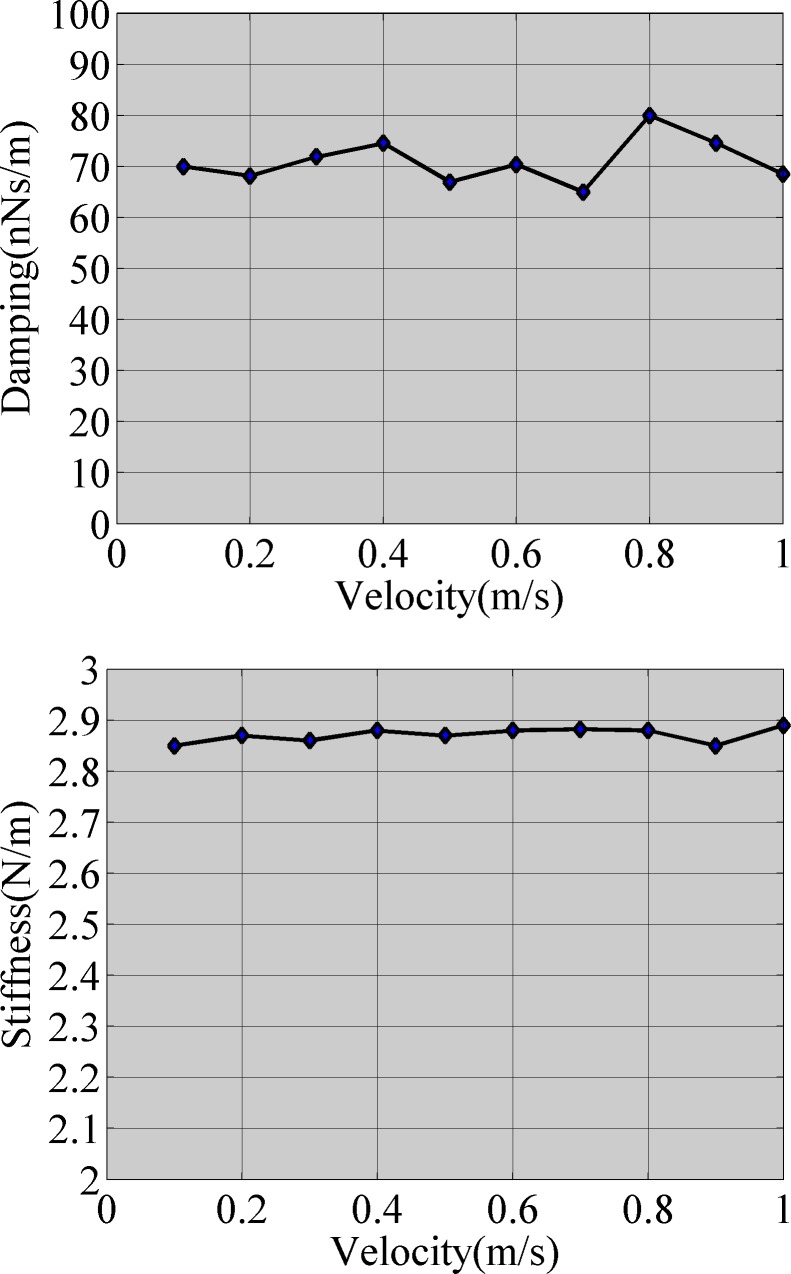
Simulation results for the fluid structure interactions: a) Viscous force versus speed, b) Effective spring constant versus speed.

The fluid model was established using the Fluent software when the boundary velocity was 0.1 m/s ~ 1 m/s (step speed 0.1 m/s) (Fig 5), and the changing curve of the boundary pressure with the boundary displacement is shown in Fig 7. According to previously mentioned method and as seen in Fig 6, we obtained the added damping and added stiffness on the micro-cantilever. Fig 8A shows the changing curve of the damping force with velocity on the micro-cantilever beam for which the slope of the curve is the added damping, *c*_*a*_. Fig 8B shows the changing curve of the added stiffness with the boundary velocity. Fig 8 indicates that the measurement methods of the damping force and stiffness can yield consistent results.

According to Fig 8, when the micro-cantilever vibrated in the air, the added damping was 6.7×10^–8^ Ns/m and the added stiffness was 2.8×10^–9^ N/m. According to the principles of vibrational mechanics, the equivalent concentrated mass of the rectangular micro-cantilever is *M* = 0.24 *m*, where *m* is the actual mass of the micro-cantilever and *M* is 1.22×10^-11^kg. Using the finite element analysis (FEA) method, the natural frequency of the micro-cantilever (*ω*_0_) was calculated as 35kHz, and based on Eq (17), *ω*_*d*_/*ω*_0_ = 99%. using numerical method, the natural frequency of the micro-cantilever (ω_0_) is calculated as 35.2 kHz and based on Eq (17), ω_d_/ω_0_ = 98.4%. Although there are certain deviations, the trend is consistent. Compared with the vacuum environment, the frequency drift always exists in the fluid environment, which is more noticeable. Compared with the simplified one-dimensional method, these results are closer to the actual measurements. However, due to excessive assumptions and simplifications, certain discrepancies occurred between the derived and the experimentally measured results. Nevertheless, the proposed method is significant for solving the FSI problems of micro-cantilevers. In particular, Eq (17) provides a method for calculating the natural frequency of the micro-cantilevers based on the FSI analysis. Furthermore, the methods for measuring the micro-cantilever parameters, such as the Young’s modulus and stiffness, can be developed. Here, the frequency drift of the micro-cantilevers was minor in the air and tremendous in other media such as water (due to the FSI). In addition, although the frequency drift is only 1–5%, the calculation error of the stiffness based on the drift frequency can be amplified and reach approximately 10% based on the relationship between the frequency and stiffness. This finding was consistent with previous reports.

## Conclusions

Here, we proposed a simplified one-dimensional method for analyzing the frequency response problems of micro-cantilevers in fluid environments. This method simplifies the vibration model of micro-cantilevers in a fluid environment into a damped vibration model for the spring-mass system. In addition, this method deduces a relationship equation for the natural vibration frequency of micro-cantilevers in fluid and vacuum environments. According to our results, an equivalent aeroelastic model was proposed to analyze the FSI problem of micro-cantilevers. In addition, a simulation analysis was conducted. The simulation results indicate that the frequency response model for micro-cantilevers that was developed based on the aeroelastic model can accurately reflect the vibration problem of micro-cantilevers in the air.
